# Phylogenetic Relationship of Phosphate Solubilizing Bacteria according to 16S rRNA Genes

**DOI:** 10.1155/2015/201379

**Published:** 2015-01-06

**Authors:** Mohammad Bagher Javadi Nobandegani, Halimi Mohd Saud, Wong Mui Yun

**Affiliations:** Institute Tropical Agriculture, Universiti Putra Malaysia, 43400 Serdang, Selangor, Malaysia

## Abstract

Phosphate solubilizing bacteria (PSB) can convert insoluble form of phosphorous to an available form. Applications of PSB as inoculants increase the phosphorus uptake by plant in the field. In this study, isolation and precise identification of PSB were carried out in Malaysian (Serdang) oil palm field (University Putra Malaysia). Identification and phylogenetic analysis of 8 better isolates were carried out by 16S rRNA gene sequencing in which as a result five isolates belong to the Beta subdivision of *Proteobacteria*, one isolate was related to the Gama subdivision of *Proteobacteria*, and two isolates were related to the *Firmicutes*. Bacterial isolates of 6upmr, 2upmr, 19upmnr, 10upmr, and 24upmr were identified as *Alcaligenes faecalis*. Also, bacterial isolates of 20upmnr and 17upmnr were identified as *Bacillus cereus* and *Vagococcus carniphilus*, respectively, and bacterial isolates of 31upmr were identified as *Serratia plymuthica*. Molecular identification and characterization of oil palm strains as the specific phosphate solubilizer can reduce the time and cost of producing effective inoculate (biofertilizer) in an oil palm field.

## 1. Introduction

Phosphorus is the least available essential nutrients where its concentrations are less than many other nutrients in soil. Since available phosphorus seldom exceeds 10 *μ*M in soil, the lack of that is common in most soil [1, 2]. Available inorganic P in soil solution is 2 *μ*M and it is several orders of size lower than that in plant tissues (5–20 mM). In the other hand, aluminum, iron, and calcium interact strongly with P and make it unavailable to plants. Also, twenty to eighty percent of phosphate in soil is in organic form [3]; it should be mineralized into inorganic form before it becomes available for plant up-taking. Several bacterial and fungal species were reported as phosphate solubilizer in crops and some of them were used as biofertilizers in agricultural fields [1].

With the aid of molecular technologies, the study of microbial ecology and understanding the place of microorganisms in society were made easy [4–7]. These days, interest for molecular identification of bacteria based on the better understanding of their biological role in keeping a sustainable biosphere has been increased [8–11]. Sequence analysis of 16S rRNA gene can find phylogenetic relationships between bacteria [11–15].

Studies have shown that diversity of microorganism in soil is large [16]. Therefore, the aim for identifying communities of microorganisms with key roles in specific soil chemical processes such as phosphate solubilization is important in soil microbiology [17–21]. The objective of this study was to isolate, identify, and characterize phosphate solubilizing bacteria from rhizosphere and nonrhizosphere of oil palm in University Putra Malaysia's soil (Serdang).

## 2. Materials and Methods

### 2.1. Soil Sampling

Soil samples were collected from oil palm plantation at University Putra Malaysia, Serdang (+2° 59′ 9.666′′ : +101° 43′ 23.5416′′). The samples were taken randomly from mature oil palm plantations (eight years) at a depth of 15–20 cm. Sampling was done in a manner which ensure that no cross contamination can occur between rhizosphere and nonrhizosphere samples. Soil samples were then analyzed to determine the chemical elements including Nitrogen [22], Phosphorus (P) [23], Potassium (K), Calcium (Ca) Magnesium (Mg) [24], Zinc (Zn), Iron (Fe), Manganese (Mn), and Copper (Cu) [25] (Table 1).

### 2.2. Isolation of Phosphate Solubilizing Bacteria

Bacterial isolates were isolated from soil samples by using root-free soil as nonrhizosphere and also by using soil which is surrounding the root of oil palm as Rhizosphere. Ten grams of soil was diluted in 95 mL of sterile water to form a serial dilution up to 10^−6^. Then, 0.1 mL of the final three dilutions was individually plated on Pikovskaya (PVK) media [26]. Bacteria which represent clearing halozone on the plates were selected and purified on PVK media [27]. The eight bacterial isolates were selected for further study based on the high performance of phosphate solubilization [28].

### 2.3. Identification of Phosphate Solubilizing Bacteria Using 16S rRNA Gene

All eight isolates were grown in Luria Broth Agar (LBA) media. Bacteria were grown at 28°C for 18 hours in a shaking incubator (Amersham Pharmacia Biotech) (200 rpm) [29]. Genomic DNA was extracted from bacterial isolates using a commercial kit (Qiagen Miniprep 27104 Matrix Technologies Cooperation, USA) according to the manufacturer's instructions. Then, DNA was stored at −20°C. The concentration and purity of the extracted DNA were determined spectrophotometrically [29]. Two universal oligonucleotide forward and reverse primers were synthesized based on the standard 16S rRNA gene sequence [30]. The primers used to amplify the 16S rDNA samples were forward primer: -5′-CCGAATTCGTCGACAACAGAGTTTGATCCTGGCTCAG-3′ and reverse primer: -5′-CCCGGATCCAAGCTTACGGCTACCTTGTTACGACTT-3′.

### 2.4. PCR Amplification

PCR amplification was performed in a total volume of 50 *μ*L mixture. It contained 5 *μ*L of 10x PCR buffer, 0.5 *μ*L of 10x dNTP, 1 *μ*L of forward and 1 *μ*L of reverse primer, 1.3 *μ*L of template DNA (10 ng), 1 *μ*L of Taq DNA polymerase, and sterile distilled water adjusted to 50 *μ*L. The tubes were subjected to 35 cycles in a thermal cycler with the following program: initial denaturation at 95°C for 3 minutes which was followed with 35-cycle consisting of denaturation at 94°C for 30 seconds, annealing at 60°C for 30 seconds, and elongation at 72°C for 2 minutes. The reaction was completed with an extension step at 72°C for 5 minutes. All samples were removed from the thermal cycler and stored at −20°C. The PCR product was determined by electrophoresis with loading 8 *μ*L of PCR product on to 1% agarose gel. The gel was stained with ethidium bromide and photographed using gel-documentation system (Hoefer PS 500XT) [29]. The PCR products of 16S rRNA gene of each sample were purified by the PCR purification kit (Vivantis, GF-PC-100, Malaysia) following the manufacturer's instructions. The 16S rRNA gene products of eight bacteria isolates were sequenced. The clean PCR product was subjected to cycle sequencings in both directions using the universal primers. The sequencing was done by ABAPRISM Dye Terminator Cycle Sequencing method (NHK Bioscience Solutions Sdn. Bhd). The nucleotide sequences were edited using the software Chromas and compared with published sequences in the National Center for Biotechnology Information, Genbank, using the BLAST software. Phylogenetical analyses of 16S rRNA gene sequences were aligned using the software CLUSTAL W 1.8. The phylogenetical analysis was conducted using the Neighbor Joining Method. Then, the output trees were performed with the software Molecular Evolutionary Genetics Analysis version 4.0 (MEGA 4) [29].

## 3. Results and Discussion

### 3.1. 16S rRNA Gene Analysis

**Figure 1:**
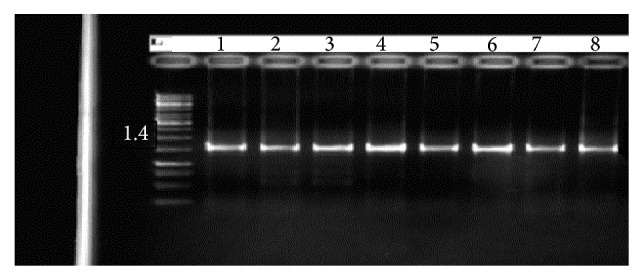
PCR products of 16S rDNA (Line 1 = 20upmnr, Line 2 = 19upmnr, Line 3 = 6upmr, Line 4 = 10upmr, Line 5 = 31upmr, Line 6 = 2upmr, Line 7 = 24upmr, and Line 8 = 17upmnr).

Two universal oligonucleotides were used to determine and identify the 16S rRNA gene for all isolates. The primer amplified the gene successfully from all of the phosphate solubilizing bacterial isolates, although there were no obvious variations in the size of rRNA gene products between the eight bacterial isolates. The size of the 16S rRNA gene product of all isolated bacteria in this study was about 1.4 Kb to the relative DNA size marker (Figure 1). The 16S rRNA gene sequence of phosphates solubilizing bacteria that was isolated from oil palm soil was compared with Genbank and received the accession numbers (Table 2). The 16S rRNA gene sequences allowed separation between isolates at the species level.

It is also important to consider that, for the identification of isolates from oil palm soil, it is not necessary to sequence the whole 1,500 bp length and thus partial sequencing can provide necessary information, even though the whole sequencing that includes the entire 1,500 bp region might be useful to distinguish between particular strains.

Comparison of the partial 16S rDNA sequence of eight isolates with Genbank database showed that they belong to two taxonomic lineages. Five isolates belonged to the Beta subdivision of* Proteobacteria*, one isolate was Gama subdivision of* Proteobacteria,* and two isolates were* Firmicutes*. Bacterial isolates of 6upmr, 2upmr, 19upmnr, 10upmr, and 24upmr were identified as* Alcaligenes faecalis*. Also, bacterial isolates of 20upmnr and 17upmnr were identified as* Bacillus cereus* and* Vagococcus carniphilus*, respectively, and bacterial isolates of 31upmr was identified as* Serratia plymuthica *(Table 2). Before, it had been reported that* Pseudomonas, Bacillus,* and* Rhizobium *strains are the most powerful and abundant strains of bacterial phosphate solubilizers [31]. Also, the genera of* Aspergillus*,* Penicillium*,* Klebsiella*,* Burkholderia,* and* Staphylococcus *are better phosphate solubilizers in Colombia oil palm plantation [13]. However,* Bacillus*,* Rhodococcus*,* Arthrobacter*,* Serratia*,* Chryseobacterium*,* Delftia*,* Gordonia,* and* Phyllobacterium* genus were better in Taiwan [32]. Furthermore, the phylogenetic diversity of phosphate solubilizing bacteria (PSB) distributed in soil of China was characterized and members of* Proteobacteria* were dominant. Most of the isolates were associated with the genera of* Burkholderia, Pseudomonas, Acinetobacter, Enterobacter, Pantoea, Serratia, Klebsiella, Leclercia, Raoultella,* and* Cedecea *[33]. Also, researcher reported that most phosphate solubilizing bacteria (PSB) in a crop/pasture rotation in Uruguay were related to the genera of Burkholderia, Acinetobacter, and genus* Pseudomonas *[34].

The 16S rRNA gene analysis revealed that all of isolates belong to the genera* Alcaligenes*,* Serratia*,* Bacillus,* and* Vagococcus*. The most often observed species of the* beta Proteobacter* genus was* Alcaligenes*. Sequences from eight isolates were completely or higher than 98% similar to other 16S rRNA sequences from database. The isolates 19upmnr, 6upmr, 2upmr, 24upmr, and 31upmr had 99% and 17upmnr isolate had 98% similarity with other isolates in gene bank database (NCBI) (Table 2).

**Figure 2:**
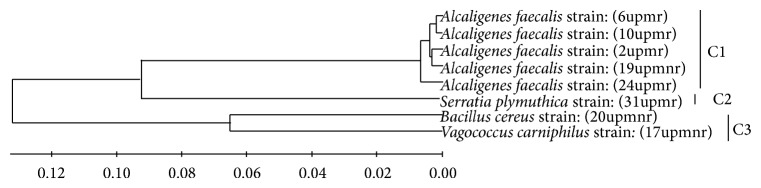
The dendrograms of cluster analysis showing the identification and relationship of UPM's PSB isolates based on 16S rRNA gene.

The phylogenetic analysis based on the partial 16S rRNA gene sequencing could classify the three main taxonomic lineages (Figure 2). The sequences obtained from* Alcaligenes*,* Serratia*,* Bacillus, *and* Vagococcus* genera formed separated branches from one another. There are three phylogeny branches that belong to* Alcaligenes* strains (6upmr, 10upmr, 2upmr, 19upmnr, and 24upmr),* Serratia* (31upmr), bacillus (20upmnr), and Vagococcus (17upmnr) (Figure 2). All isolates form three distinct clusters based on near full-length 16S rRNA gene sequence analysis. Cluster C1 belongs to* Alcaligenes *strains that were isolated from rhizosphere and nonrhizosphere environment at Serdang (UPM) soil. Comparison of sequences revealed a greater genetic diversity in* Alcaligenes* strains. Isolate 31upmr from UPM rhizosphere formed separate cluster (C2), while the other showed close relationship with each other (cluster C3). These results suggested that the* Alcaligenes* genus grouped at cluster C1 was diverse and needed another molecular marker to distinguish between them.

It suggested that a physiological stress or effects of environment led to selection of less diverse communities in bacterial populations and general suppression of high solubilizing activity. The results showed the differences in the isolate's sequences in two areas of sampling, while most isolated bacteria from nonrhizosphere and rhizosphere belonged to* Alcaligenes* species. Rhizosphere bacteria were more diverse because of the population compared with nonrhizosphere.

## 4. Conclusion

In conclusion, conservation region in the 16S rRNA gene sequence could identify all isolates of phosphate solubilizing bacteria isolated from oil palm soil successfully. This sequence can serve as a good molecular chronometer for identification of phosphate solubilizing bacteria with no previous knowledge. The degree of gene conservation is considered to be a significant part of cell identification. This study, also, shows that partial sequencing can provide statistically valid measurements for evolutionary distances of phosphate solubilizing isolates. Assigning a numerical value to the rate of change in phosphate solubilizing isolates can make taxonomic groups for all isolates. Furthermore, it shows that the rate of change in phosphate solubilizing isolates can be different at University Putra field. Finally, no gene has shown as broad applicability over all the taxonomic groups as the 16S rRNA gene. Thus, if the objective would be to identify an unknown isolates, the 16S rRNA gene sequence is an excellent and extensively used choice.

## Figures and Tables

**Table 1:** Soil analysis.

Sample		%		*μ*g/g							
	pH	C	N	P	K	Ca	Mg	Cu	Fe	Mn	Zn
UPMNR	5.4^a^	1.6^b^	0.1^b^	39.8^b^	38^b^	402^a^	81^b^	0.89^a^	0.89^a^	3^b^	2^b^
UPMR	5.0^b^	5.8^a^	0.43^a^	45.3^a^	129^a^	337^b^	102^a^	0.52^b^	0.52^a^	10^a^	6^a^

In each column followed by different letters are significantly different (*P* < 0.05) according to ANOVA test performed with SPSS 10.1 Software.

UPMNR: UPM nonrhizosphere and UPMR: UPM rhizosphere.

**Table 2:** Identification and relationship of PSB isolates based on 16S rDNA marker data.

Name	Kingdom	Phylum	Class	Order	Family	Genus	Species	Accession number (NCBI)
17upmnr	Bacteria	*Firmicutes *	*Bacilli *	*Lactobacillales *	*Enterococcaceae *	*Vagococcus *	*V. carniphilus *	KJ783452
20upmnr	Bacteria	*Firmicutes *	*Bacilli *	*Bacillales *	*Bacillaceae *	*Bacillus *	*B. cereus *	KJ729602
19upmnr	Bacteria	*Proteobacteria *	*Betaproteobacteria *	*Burkholderiales *	*Alcaligenaceae *	*Alcaligenes *	*A. faecalis *	KJ748593
6upmr	Bacteria	*Proteobacteria *	*Betaproteobacteria *	*Burkholderiales *	*Alcaligenaceae *	*Alcaligenes *	*A. faecalis *	KJ729608
2upmr	Bacteria	*Proteobacteria *	*Betaproteobacteria *	*Burkholderiales *	*Alcaligenaceae *	*Alcaligenes *	*A. faecalis *	KJ748586
10upmr	Bacteria	*Proteobacteria *	*Betaproteobacteria *	*Burkholderiales *	*Alcaligenaceae *	*Alcaligenes *	*A. faecalis *	KJ748585
24upmr	Bacteria	*Proteobacteria *	*Betaproteobacteria *	*Burkholderiales *	*Alcaligenaceae *	*Alcaligenes *	*A. faecalis *	KJ748587
31upmr	Bacteria	*Proteobacteria *	*Gammaproteobacteria *	*Enterobacteriales *	*Enterobacteriaceae *	*Serratia *	*S. plymuthica *	KJ729609
